# Multicomponent Behavior Change Technique Intervention for Caregivers of People With Alzheimer Disease and Related Dementias: Protocol for a Single-Arm, Personalized Behavioral Trial to Disrupt Sedentary Time

**DOI:** 10.2196/82857

**Published:** 2026-06-09

**Authors:** Danielle Miller, Sherene Lambert, Luis Jordan, Mark J Butler, Liron Sinvani, Alexandra Perrin, Ying Kuen Cheung, Karina W Davidson, Ashley M Goodwin

**Affiliations:** 1 Northwell, 2000 Marcus Ave, Suite 300, New Hyde Park, NY, 11042, United States, 646-995-8958; 2 Mailman School of Public Health at Columbia University, New York, NY, United States

**Keywords:** behavior change technique, sedentary behavior, habit formation, caregivers, Alzheimer disease and related dementias

## Abstract

**Background:**

Sedentary behavior is associated with negative health outcomes. High levels of sedentary behavior are common among Alzheimer disease and related dementias (ADRD) caregivers already at risk of other adverse health effects, yet few interventions target sedentary behavior within this population. There is a need for trials intended to reduce time spent sedentary, which may be achievable by increasing the frequency of disruptions to sedentary time. Remotely delivered behavior change techniques (BCTs) may be effective for disrupting sedentary behavior in this population through short bursts of walking, although it is unclear how BCTs promote this behavior and potentially act via the hypothesized mechanism of behavioral automaticity.

**Objective:**

The goal of the trial is to examine whether a significant proportion of ADRD caregivers (≥50%) receiving an SMS text message–delivered BCT intervention form a habit to engage in hourly walking 4 times per day, with the broader objective of disrupting sedentary time in this population.

**Methods:**

This trial is a 12-week, decentralized, single-arm, National Institutes of Health Stage II behavioral trial. The trial will deliver a personalized, multicomponent BCT intervention to disrupt time spent sedentary by encouraging forming a habit of hourly walking among caregivers of persons with ADRD via the key mechanism of behavior change behavioral automaticity. The intervention includes 4 daily SMS text message–delivered BCT components previously used in interventions to disrupt sedentary behavior—Goal setting, Action planning, Prompts/cues, and Self-monitoring. Formation of an hourly walking habit is the primary outcome and will be defined as walking an additional 250 steps or more per hour for the same 4 consecutive hours as set up in a personalized walking plan on 7 consecutive days. Secondary outcomes include evaluating associations between habit formation and behavioral automaticity, and between longitudinal behavioral automaticity and habitual hourly walking over time. Additionally, heterogeneity of treatment effects will be evaluated. Exploratory analyses will examine potential moderating variables that may influence the intervention effect. The trial uses digital enrollment strategies, SMS text message intervention delivery, passive data collection via Fitbit (Google) devices, and online survey assessments to collect data remotely.

**Results:**

This study was funded by the National Institute on Aging in June 2024. Recruitment and data collection began in March 2025. As of August 2025, 40% (n=40) of the planned sample has been enrolled. Data collection is expected to be complete by June 2026. Data analysis and publication of results are expected by Fall and Winter 2026, respectively.

**Conclusions:**

Results will have the potential to advance knowledge about the effectiveness of BCTs to form a habit of hourly walking and may provide opportunities for future public health impact to promote physical activity in caregivers of those living with ADRD.

## Introduction

The Sedentary Behavior Research Network defines sedentary behavior as “any waking behavior characterized by an energy expenditure ≤1.5 metabolic equivalents, while in a sitting, reclining or lying posture” in adults ≥18 years of age [1]. Examples of sedentary behavior are sitting while working, watching television, or using the computer [2]. According to accelerometer data, US adults spend anywhere from 55% to 86% of their time engaging in sedentary behavior [3-5]. Certain populations are more sedentary than others. Sedentary behavior is particularly common among Alzheimer disease and related dementias (ADRD) caregivers, who make up 26% of the caregiver population in the United States and spend nearly 80% of their waking hours sedentary [6,7]. More time spent sedentary is associated with a higher risk of mortality and morbidity, independent of risks resulting from low physical activity levels [8-11]. There is strong evidence that high levels of sedentary behavior are significantly associated with incidence and mortality due to cardiovascular disease, with incidence of type 2 diabetes, and potentially with incidence of certain types of cancers [11]. High sedentary groups also have a 22% higher dementia risk than low sedentary groups [12,13]. For caregivers, this is particularly concerning given their already elevated risk of negative mental and physical health, poorer quality of life, and worse financial well-being outcomes [14-23]. Given the goal of the National Plan to Address Alzheimer’s Disease to support individuals living with ADRD and their families through caregiver health and well-being promotion, the ADRD caregiver population is a key population for quality interventions targeting sedentary behavior [7,24]. This investment is potentially valuable for both caregivers and those for whom they provide care.

Despite the established need, few interventions in recent literature focus on disrupting sedentary behavior. A meta-analysis of mobile health interventions for physical activity and sedentary behavior in older adults found that only 1 of 33 reviewed studies was designed to target sedentary behavior [25]. Thus, researchers have called for more controlled intervention trials that increase the frequency of interruptions to sedentary time throughout the day, and this is particularly important in targeted populations like caregivers [26-29]. As previous studies have shown that ADRD caregivers prefer to engage in shorter duration exercise (namely, 10-min bouts), incorporating small bouts of behavior changes (eg, walking) into the daily routines of caregivers who are inactive may be a feasible strategy to disrupt sedentary time [29-32]. Some evidence suggests that compared with complex tasks, small, simple actions like this may result in habit formation [33].

Habit formation is theorized to be possible once a stable context (such as time of day or location) allows for a response to repeat enough that the habit is then activated by cues in that context, which can override intention [34-36]. As such, habits are believed to be relatively resistant once formed so long as context is stable [34,35]. Multiple studies have demonstrated successful behavior change with habit formation [37]. Health interventions using behavior change techniques (BCTs) that are rooted in cognitive strategies like reminders and implementation planning are a promising direction for forming habits when paired with stable contexts [35,36]. This is particularly relevant for health behaviors like exercise that require longer-term repetition to result in observable benefits [36].

Behavior change techniques are observable, replicable, irreducible intervention components, thought to be the “active ingredients” to facilitate behavior change [38]. Using BCTs that support the formation of a behavioral habit, such as disrupting sedentary time, may be effective for behavior change [39]. Four BCTs in particular (Goal setting, Action planning, Prompts/cues, and Self-monitoring of behavior) have been previously used in interventions to disrupt sedentary behavior [40-46]. However, there remains a lack of evidence for *how* BCT interventions promote behavior, which is integral to understanding how to optimize and adapt interventions for special populations [47-52]. Given the overlapping features between automaticity and habit, behavioral automaticity is believed to be a possible mechanism of behavior change (MoBC) [37,39,53]. This trial focuses on forming a habit and testing the possible MoBC that causes habit formation using a validated survey assessment; automaticity’s role in *maintaining* a habit may require additional exploration.

Engaging caregivers in longitudinal clinical trial interventions can be challenging [54]. Clinical trials that successfully enroll caregivers exist, although attrition has been a limitation in some [55]. There is a clear need for trials that have considered the unique needs and circumstances of the ADRD caregiver population, such as by using remote trial designs and tailoring for caregivers’ schedules while using high-quality designs rooted in established theories [36,56,57]. Thus, this 12-week remotely delivered decentralized behavioral trial will test the efficacy of a personalized 4-BCT intervention package to form a habit of physical activity as a means of disrupting time spent in sedentary behavior among caregivers of persons with ADRD. This trial uses fully remote strategies for recruitment and enrollment, delivery of text-message interventions, collection of wearable device data via Fitbit (Google) devices, and collection of electronic survey assessments. The trial is informed by habit formation theory, and tests if behavioral automaticity is the key MoBC. The goal of the trial is to examine whether the described BCT intervention is associated with habit formation in ADRD caregivers by walking 4 times a day, reducing levels of overall sedentary time. This trial also addresses gaps in the literature by examining the association between the BCT intervention and habit formation, measured using the potential key MoBC of the walking intervention, behavioral automaticity. Other secondary hypotheses include the timing of behavioral automaticity change and its impact on habit, and the presence of heterogeneity of treatment effects (HTEs) for habit formation and changes in behavioral automaticity. We will also explore moderators of the intervention in exploratory analyses.

## Methods

### Study Design

**Figure 1.**
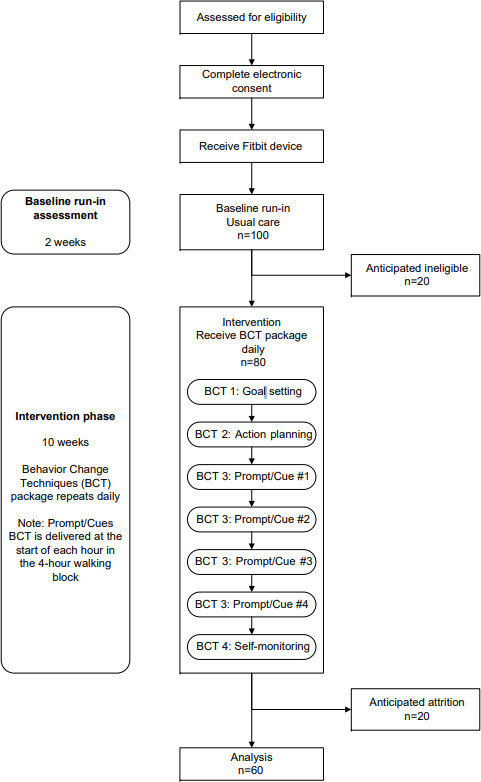
Participant timeline. Overview of 12-week trial timeline. BCT: behavior change technique.

This fully remote, single-arm, National Institutes of Health (NIH) Stage II decentralized behavioral trial delivering a multicomponent, personalized BCT intervention will enroll 100 caregivers of persons living with ADRD into a 12-week trial comprised of a 2-week baseline period and a 10-week intervention period [58]. The single-arm design of this trial was used to minimize sample size requirements and ensure all eligible caregivers were able to participate in the intervention. Participants will be provided with a Fitbit device for continuous activity tracking and electronic surveys delivered to their smartphones. Fitbit data will be retrieved directly via an Application Programming Interface call. All survey data will be collected and managed using REDCap (Research Electronic Data Capture), a secure, web-based software platform designed to support data capture [59,60]. An overview of the 12-week trial timeline can be seen in Figure 1.

### Study Population

Adults (18‐85 y of age), English- and Spanish-speaking caregivers who self-report low levels of physical activity or walking, and those who live in the United States will be recruited to participate. Formal (defined as paid, professional caregivers) and informal (defined as unpaid caregivers) caregivers of persons with ADRD living in the United States will be eligible. Additional inclusion and exclusion criteria are outlined in Textbox 1. Despite our exclusion criteria of smartphone, internet, and email access, we do not anticipate any challenges enrolling a diverse sample. Cross-sectional surveys indicate 91% of Americans own a smartphone, and 82.5% of US caregivers for older adults have smartphones [61,62]. Furthermore, 83.2% of caregivers for adults access the internet and/or use email from a mobile device [63].

Textbox 1. **Inclusion and exclusion criteria.**

**Inclusion criteria**

- Identify as a caregiver (formal or paid, or informal or unpaid) for persons with Alzheimer Disease or Alzheimer disease and related dementias
- Age ≥18 and ≤85 years
- Speak English or Spanish as primary language
- Self-report low levels of physical activity or walking

**Exclusion criteria**

- Individuals who self-report having been informed by a clinician it is medically or physically unsafe to engage in a walking intervention
- Does not own or cannot regularly access a smartphone capable of receiving SMS text messages or accessing the internet
- Does not own or have access to an email address
- Lives outside the United States

### Recruitment and Consent

Digital recruitment will include email blasts, social media postings, and advertisements in newsletters. Social media platforms, such as Reddit and Facebook (Meta), will also be used to recruit potential participants. In-person recruitment will be conducted in Northwell Health community settings as well as at community events outside of the Northwell Health system. Other recruitment methods may include using the electronic health record to identify individuals with ADRD caregiver status, emails to individuals who previously expressed interest in future research, studies at the Institute of Health System Science, trial listing platforms, and word of mouth.

All study materials (eg, recruitment materials, consent forms, surveys, BCT content, text messages, and other communications) have been translated into US Spanish by a third-party with International Organization for Standardization (ISO) certifications for Quality Management System (ISO 9001:2015) and Language Industry Certification System (ISO 17100:2015), and approved by the Northwell Health Institutional Review Board (IRB). Survey measures for which there is already a validated version in Spanish will be used (described in detail in the Methods section); otherwise, the translated survey from the third-party vendor will be used. At least 2 native Spanish-speaking research team members will review the third-party translations to ensure semantic (eg, ensuring terms and phrases make sense) and idiomatic (eg, ensuring local equivalents for colloquial terms are used) accuracy and dispute any concerns with the third-party translators directly to achieve final translated products. Participants will be asked during screening which language they would prefer to receive study information in. Consenting coordinators who are fluent in the language participants choose (eg, English or Spanish) will conduct research conversations to answer questions and provide clarification about the study.

Interested individuals will be directed by QR code or link to a digital landing page in REDCap. The digital landing page will outline the study’s purpose and procedures, along with an informational video summarizing the study’s purpose and procedures in their preferred language. Interested individuals will be asked to complete a HIPAA (Health Insurance Portability and Accountability Act) authorization form, a demographic survey required by the funder, and a screening questionnaire to assess their eligibility according to inclusion and exclusion criteria. Study staff will review responses to confirm participant eligibility before sending them the electronic consent form. Those who are ineligible will be notified immediately via an automated message within REDCap. For those who are eligible, study staff will send a link to an electronic consent form to review and sign, which includes a short 3-question assessment to evaluate participant comprehension of the study and consent process. Trained, consenting coordinators (SL and LJ) will review the signed informed consent form for completeness and accuracy and document an enrollment note. Study recruitment began in March 2025.

### Prebaseline Period

**Figure 2.**
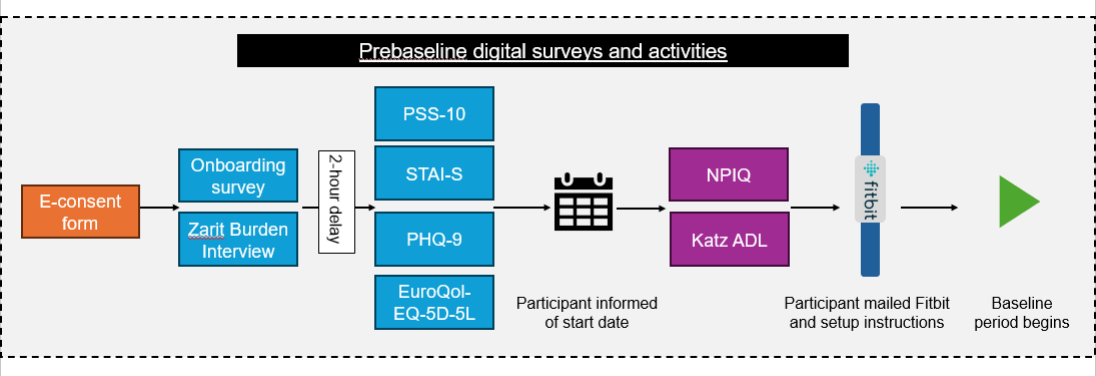
Prebaseline digital surveys and activities for consented participants. Katz ADL: Katz Activities of Daily Living; NPIQ: Neuropsychiatric Inventory-Questionnaire; PHQ-9: Patient Health Questionnaire-9; PSS-10: Perceived Stress Scale; STAI-S: State-Trait Anxiety Inventory – Short Form.

After consent, participants will be led to complete a series of digital surveys in REDCap (Figure 2). An onboarding survey will include questions about caregiving and caregiving responsibilities (eg, number of care recipients, relation to person with ADRD, primary caregiver status, length of time providing care, hours providing care per week, and living situation), care recipient characteristics (eg, time since diagnosis and demographic characteristics of person with ADRD), and questions to personalize the study to the caregiver (eg, shipping address, preferred times for contact, and preferred start date). Additionally, participants will complete a digital assessment of caregiver burden (Zarit Burden Interview [64]). After a 2-hour break, participants will receive an SMS text message via a Twilio integration in REDCap (delivered using the Automated Survey Invitations application) with a link to complete a sequence of digital assessments of stress (Perceived Stress Scale [PSS-10] [65,66]), anxiety (State-Trait Anxiety Inventory – short form [STAI-S] [67]), depressive symptoms (adapted version of the Patient Health Questionnaire-9 [PHQ-9] depression module [68]), and quality of life (EuroQol-EQ-5D-5L [69-71], described in more detail in this section). Consenting coordinators will confirm and assign an upcoming baseline start date to participants on a rolling basis. After participants are informed of their start date, they will be mailed a Fitbit device. They will also be automatically sent an SMS text message with a link to REDCap to complete a final set of digital assessments (delivered using the Automated Survey Invitations application) before the baseline period begins—Neuropsychiatric Inventory-Questionnaire [NPIQ] to assess care recipient neuropsychiatric symptoms and associated caregiver distress and Katz activities of daily living (ADLs) to assess care recipients’ ability to perform 6 basic functions independently [72-75]. Participants will receive regular automated SMS text message reminders via REDCap (delivered using the Automated Survey Invitations application) to complete any surveys that are incomplete.

### Baseline Period

On the participant’s assigned start date, they will begin a 2-week baseline period to assess adherence to the study protocol and preintervention behavioral automaticity. Participants will not receive any BCTs during the baseline period. Instead, they will engage in their usual level of activity, wear the Fitbit device continuously, and complete weekly SMS text message–delivered surveys via REDCap (delivered using the Automated Survey Invitations application) consisting of the 4-item Self-Report Behavioral Automaticity Index (SRBAI) [76] to assess the MoBC and a single-item assessing overall health from the EuroQol EQ-5D-5L.

**Figure 3.**
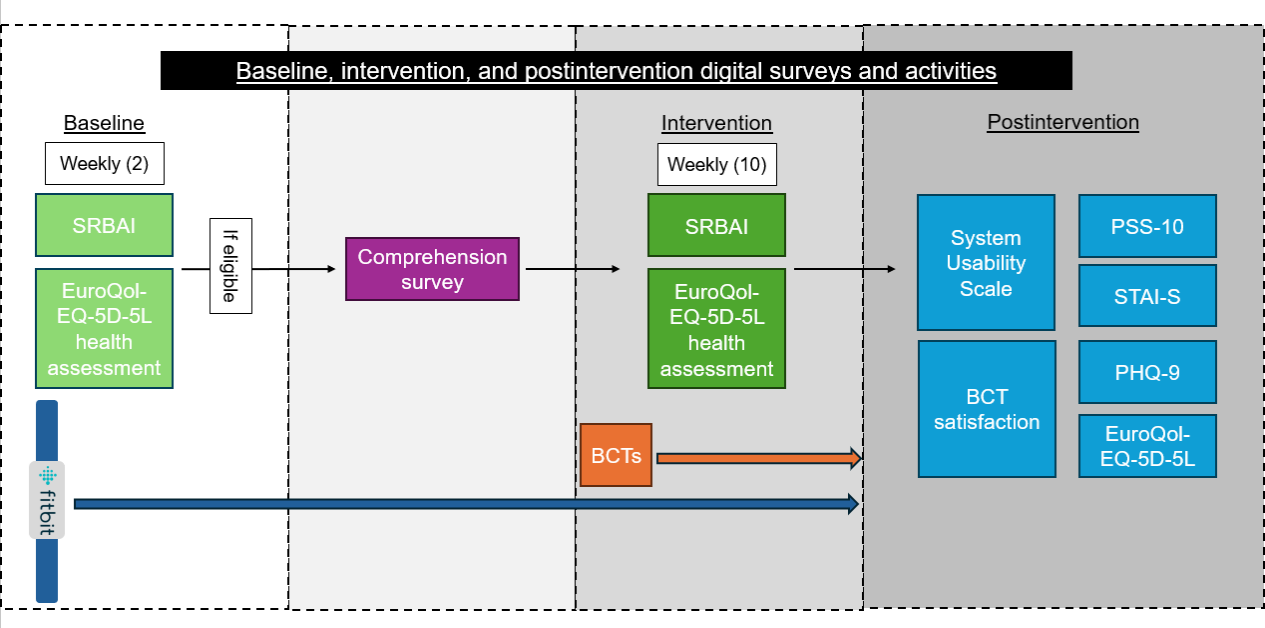
Timeline of baseline, intervention, and postintervention digital surveys and activities. BCT: behavior change technique; PHQ-9: Patient Health Questionnaire-9; PSS-10: Perceived Stress Scale; SRBAI: Self-Report Behavioral Automaticity Index.

To be eligible to continue into the 10-week intervention period, participants must wear their Fitbit for at least 10 hours each day for at least 12 of the 14-day baseline period and must complete one of the 2 weekly surveys. We have successfully achieved desired samples without more-than-expected baseline attrition using similar adherence thresholds used in other remotely delivered behavioral trials [77-80]. Participants who do not meet adherence requirements for the 2-week baseline period will be removed from the study. Figure 3 outlines a timeline of baseline, intervention, and postintervention digital surveys and activities for participants eligible after baseline.

Before the intervention period begins, participants who successfully meet or exceed the adherence requirements for the 2-week baseline period will complete a short, digital comprehension survey after reviewing an infographic illustrating the intervention phase of the study. Participants who demonstrate understanding by completing the true or false questions accurately and agree to continue to the intervention period will select their preferred 4-hour walking block to walk 250 more steps per hour each day. Participants will have up to 3 days between their baseline period end date and intervention period start date to complete this survey. Participants who do not complete the survey will be contacted by the study team at least 3 times before they are determined to be ineligible to proceed to the intervention period.

### Intervention Period

During the 10-week intervention period, participants will continue to wear their Fitbit and complete the same weekly survey (SRBAI and a single-item assessing overall health from the EuroQol EQ-5D-5L) as during the baseline period. Additionally, participants will receive an automated multicomponent BCT intervention package consisting of 7 SMS text messages daily via REDCap (delivered using the Alerts and Notifications application) with 4 BCTs to promote a walking habit of taking an additional 250 steps per hour for the same 4 hours each day.

### BCT Intervention

#### Overview

**Figure 4.**
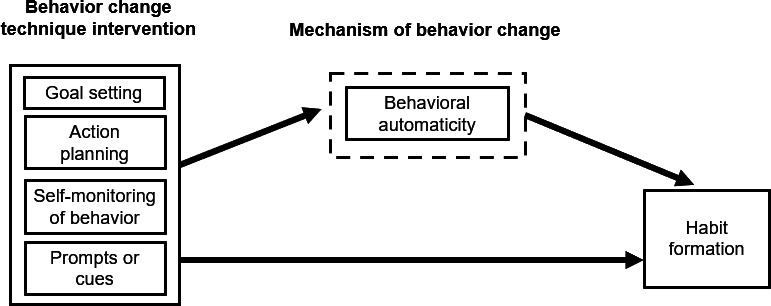
Conceptual model linking the behavior change technique intervention, mechanism of behavior change, and primary outcome of habit formation.

Participants will receive 4 BCTs daily (Goal setting, Action planning, Prompts/cues, and Self-monitoring of behavior) to encourage walking 250 steps per hour for 4 consecutive hours per day. Each BCT is delivered as an individual SMS text message to the participant’s cell phone. Participants are not required to respond to the BCT text message, as REDCap is not equipped to receive and store data from SMS text message responses, and to reduce perceived participant burden. These BCTs have all been previously used in interventions to reduce sedentary behavior [40-45]. One study has shown the combination of Action planning and Self-monitoring to be especially effective in reducing sedentary behavior time [40]. These are the BCTs hypothesized to increase the probability of successful habit formation [37]. Figure 4 presents a conceptual model linking the 4 intervention components of the BCT intervention, the MoBC behavioral automaticity, and the primary outcome of habit formation.

Some previous research has shown that caregivers spend 12 (SD 2.8) hours being sedentary per day and receive an average of 7.6 (SD 1.2) hours of sleep per night [6,46]. Spending more than 7 hours per day in a nonsedentary position is associated with a reduced likelihood of developing metabolic syndrome [47]. Furthermore, an additional 250 steps per hour for 4 hours translates to an additional 1000 steps per day, which has been found to have beneficial effects on physical and psychological health in previous research [48-50]. We deduce that 4.4 hours of caregivers’ waking hours are nonsedentary (24-h d –12 h sedentary per d – 7.6 h of sleep per night). Successful disruption of sedentary behavior with 1000 steps per day spread across a 4-hour block (250 steps per h) would result in caregivers achieving an average of over 8 hours of nonsedentary hours per day (4.4 nonsedentary h+4 additional nonsedentary h with successful disruption). This meets the average number of hours per day (>7 h) spent in a nonsedentary position to reduce metabolic syndrome while also meaningfully increasing daily steps and in a feasible manner (eg, in short bursts that disrupt sedentary time).

Below is a definition of each BCT and the delivery timing [38]:

#### 1.1 Goal Setting (Behavior)

Goal Setting is defined as encouraging an individual to set or agree on a goal, defined in terms of the behavior to be achieved (eg, take 1000 steps today). Caregivers will set a goal of taking 250 steps each hour for 4 consecutive hours, constituting a 4-hour walking block. They will receive the Goal Setting BCT daily via SMS text message at their preferred time to receive SMS text messages to encourage them to set a goal for their next 4-hour walking block.

#### 1.4 Action Planning

Action planning is defined as a prompt for detailed planning of the performance of the behavior that includes context, frequency, duration, and intensity. Caregivers will identify a plan to facilitate hourly movement during the day. They will receive the Action Planning BCT daily via SMS text message at their preferred time to receive SMS text messages to develop a plan for taking steps during their next 4-hour walking block.

#### 7.1 Prompt or Cues

Prompt/Cues is defined as introducing or defining environmental or social stimulus with the purpose of prompting or cueing the behavior, normally occurring at the time or place of performance. Caregivers will receive the Prompts/Cues BCT 4 times daily via SMS text message, one at the start of each hour during the 4-hour walking block, to prompt walking of at least 250 steps per hour.

#### 2.3 Self-Monitoring of Behavior

Self-monitoring is defined as monitoring and recording behavior. By monitoring information concerning physical activity (ie, number of steps taken), individuals can observe their progress over time. Caregivers will receive the Self-Monitoring BCT daily via SMS text message approximately 30 minutes after the end of their 4-hour walking block.

**Figure 5.**
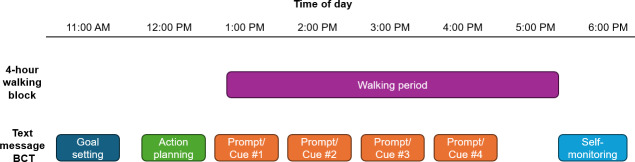
Sample personalized daily behavior change technique schedule. Sample schedule for a caregiver participant with a preferred 4-hour walking block of 1‐5 PM. BCT: behavior change technique.

Table 1 illustrates the BCT text message prompts participants will receive in English and Spanish. A sample personalized daily BCT schedule is presented in Figure 5.

**Table 1.** Description of BCTs^a^ and SMS text messages, in English and Spanish.

BCT	Language	
	English	Spanish
1.1 Goal Setting (Behavior)	Take a moment to set a goal to walk at least 250 extra steps each hour during your next walking time between *[walking period start time]* and *[walking period end time]*.	Tómese un momento para la fijación de un objetivo para caminar al menos 250 pasos más cada hora durante su próxima caminata entre *[walking period start time]* y *[walking period end time]*.
1.4 Action Planning	Think about where, when, and for how long you will walk to take at least 250 extra steps each hour during your next walking time between *[walking period start time]* and *[walking period end time]*.	Piense en dónde, cuándo y durante cuánto tiempo caminará para dar al menos 250 pasos más cada hora durante su próxima caminata entre *[walking period start time]* y *[walking period end time]*.
2.3 Self-Monitoring of Behavior	Take a moment and reflect: did you stand up and walk at least 250 extra steps each hour between *[walking period start time]* and *[walking period end time]* today? You do NOT need to respond to this message.	Tómese un momento y reflexione: ¿se levantó y caminó al menos 250 pasos más cada hora entre *[walking period start time]* y *[walking period end time]* hoy? NO es necesario que responda a este mensaje.
7.1 Prompts/Cues	It’s time to take at least 250 extra steps this hour.	Es hora de dar al menos 250 pasos adicionales esta hora.

a BCT: behavior change technique.

The research team will monitor for fidelity during the implementation of intervention procedures. After a participant passes the baseline period and completes the short, digital comprehension survey containing questions on their preferred 4-hour walking block, trained research team members (SL and LJ) will review their preferences and manually enter the preferred times into a research team–facing instrument in REDCap. A second trained research team member (SL or LJ) will compare the entered preferred times in the research team–facing instrument with the participant’s comprehension survey for accuracy. If accurate, the research team member will submit the research team–facing instrument, triggering an automation in REDCap (delivered using the Alerts and Notifications application) that will schedule the daily BCT intervention for the length of the intervention period according to the personalized times entered. Additionally, a trained research team member (SL or LJ) will review the REDCap Alerts and Notifications log for each participant on a weekly basis to ensure the daily multicomponent BCT intervention was sent and at the correct times. Participants may receive additional SMS text messages during the intervention period to remind them to sync their device with the Fitbit app on their phone at least every 2 days, to charge their Fitbit device when the battery is at or below 25%, and to answer incomplete surveys. At completion of the 10-week intervention period, participants will be asked to complete postintervention assessments of caregiver burden (Zarit Burden Interview), stress (PSS-10), anxiety (STAI-S), depressive symptoms (PHQ-9), and quality of life (EuroQol EQ-5D-5L). They will also be asked to complete end-of-study surveys assessing the usability and acceptability of the trial (System Usability Scale) [81-83], and satisfaction with the BCT text messages and overall trial. Trial completion will be defined as submission of these end-of-study surveys.

Participants will have an option to fully or partially (defined as passively providing Fitbit data only for the remainder of the trial) withdraw from the research at any point by reaching out to the study team as detailed in the informed consent form. Fully withdrawn participants will receive a short survey asking for the reason they are opting to withdraw from the research, and given the opportunity to complete the end-of-study surveys that all other participants complete at the end of the intervention period.

### Ethical Considerations

This study underwent institutional review and was approved as a no-greater-than-minimal-risk trial by the Northwell Health IRB (IRB #24‐0042) in February 2024 (protocol version 1, dated February 13, 2024). The protocol was developed by the principal investigator AMG, coinvestigator MJB, coinvestigator LS, and study manager DM (all affiliates of Northwell Health). All procedures will be followed in accordance with the ethical standards of the Northwell IRB. All amendments to the protocol, consent form, and other trial-related documents will be submitted for approval to the Northwell Health IRB and communicated to participants of the research at the discretion of the Northwell Health IRB.

All participants will provide authorization and informed consent to enroll in the research described in detail in the Recruitment and Consent subsections. During the consent process, participants will be informed of the voluntary nature of the research and the option to withdraw from the research at any time.

The principal investigator (AMG) will be responsible for ensuring safe data management practices. Several protective measures will be taken to safeguard participant information. All survey data collected via REDCap will be stored automatically in REDCap, which is restricted by user rights controlled by the study manager (DM). All Fitbit data collected via a Fitbit wearable device will be automatically pulled via Fitbit’s Application Programming Interface and securely stored in the Northwell Health Research Data Lake. Trial records that identify participants will be maintained in a secure, password-protected, HIPAA-compliant database. These records will be kept private and only accessible by trained trial staff approved by the Northwell IRB. Only necessary information about participants will be shared with approved parties stated in the informed consent form (such as the trial funder, upon request). Participants will not be identified in trial records or publications disclosed outside Northwell Health. Data will be shared only according to parameters described in the informed consent form and in coded format (ie, we will separate the participant’s personal information [name, address, and cell phone number] from all other information provided). The key to participants’ identifiable information will be stored in the secure, password-protected, HIPAA-compliant database.

The principal investigator (AMG) will be responsible for ensuring participant safety on a daily basis. An independent local safety monitor, who is a board-certified physician, will monitor participant safety and evaluate the progress of the study according to the trial data and safety monitoring plan approved by the funder. This trial proposes a nonsystematic collection method of reported events to determine if an adverse event has occurred, and to determine next steps, if any, for reporting. Events reported by participants that meet the definition of an adverse event will be collected in electronic format using REDCap. The local safety monitor will receive regular safety reports from the principal investigator (AMG) and trial team detailing reported adverse events and will be asked to identify any issues or concerns with the interpretation and management of adverse events to ensure new safety concerns with the conduct of this research are addressed.

Participants will be paid a US $200 ClinCard (reloadable prepaid pay card) after completing the end-of-study surveys and will be able to keep the Fitbit (valued at about US $160) as a “thank you” for their participation. Additionally, participants who are in the intervention period will be entered in a random weekly lottery where one participant is selected. If the selected participant was adherent (ie, wore their Fitbit for at least 10 h/d during 6 of 7 d during that week and answered the weekly survey from that week), they will be paid a US $150 ClinCard prize. Adherence to the intervention is not a condition for the lottery. Participants who are selected for the lottery but were not adherent and therefore did not win the prize will be told as such and be given instructions on how they can become eligible for the prize next week. All other participants will be notified of their adherence the week before, and a reminder of how they can be eligible for future lottery prizes. During the 10-week intervention period, participants may be selected and awarded the lottery prize up to 10 times. Participants who are fully withdrawn or partially withdrawn from the study will be made aware that they are no longer eligible for the end-of-study compensation, but will be able to keep the study-provided Fitbit and be paid for any lottery prizes they won before withdrawing.

### General Statistical Analytical Approach

Baseline measurements will be summarized with descriptive statistics, including mean, median, and SD for continuous variables (eg, age) and proportions for categorical variables (eg, sex). In regression models, all coefficients will be estimated with 95% CIs, and for the point estimates, 2-sided *P* values will be used. All analyses will be conducted as intent-to-treat using all available data for participants who receive the intervention. We will do multiple imputation using within-subject data during the intervention as a sensitivity analysis to handle missing data. For analyses of the primary outcome, missing step data will be assessed conservatively and will be marked as having the absence of the target behavior (eg, walking 250 steps each h during their preidentified, preferred 4-h period each day for 7 consecutive d) for that day or days.

### Primary Outcome and Analysis

**Figure 6.**
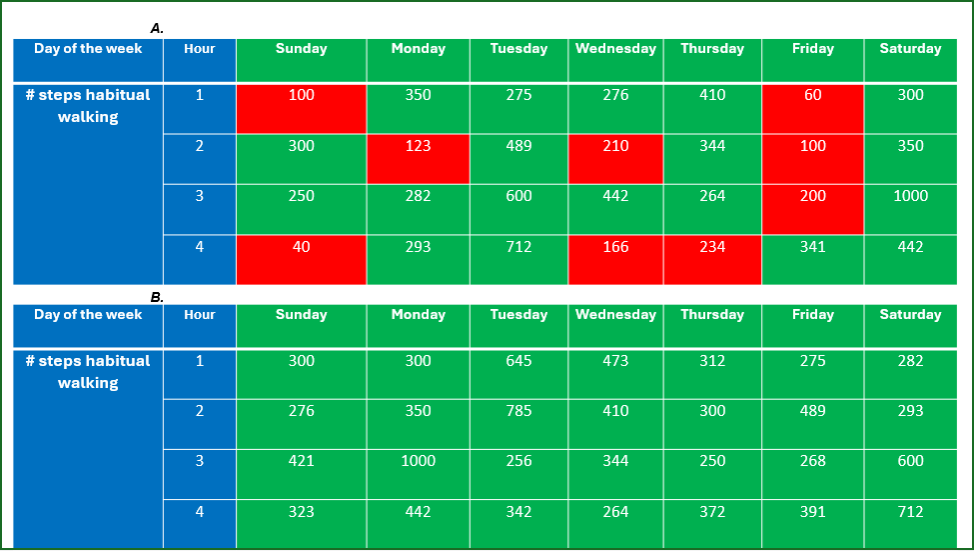
Primary outcome – formation of habitual hourly walking. (A) No habitual hourly walking, and (B) Habitual hourly walking. Example of Fitbit step counts for a participant who did not achieve habit formation (A) and a participant who did achieve habit formation (B).

The primary outcome is a binary indicator of habit formation. The goal is to identify whether a significant proportion of caregivers (≥50%) develop habitual hourly walking, thereby disrupting the time they spend sedentary. A participant will have successfully formed a habit of walking if they attain walking 250 steps each hour during their preidentified, preferred 4-hour period each day for 7 consecutive days during the 70-day intervention period (Figure 6). We used this definition of habit to reflect behavioral consistency in physical activity performance. Consistency of physical activity behavior has been found to be a strong correlate of habit in previous research [84]. To ensure that our definition of habit does not bias results, we will conduct sensitivity analyses using 10-day, 14-day, and 21-day periods in addition to the 7-day period used for our primary outcome. We will use a null hypothesis rate of 30% and will compare the achieved proportion of habit formation versus this null level, using a 1-sample binomial test with a 2-sided α at 5%. The rate of habit formation will be summarized using the observed proportion along with a 95% CI. We will also use Kaplan-Meier curves to identify estimates of the time-to-event for habit formation in the sample.

### Secondary Outcomes and Analysis

One of the secondary hypotheses of this trial is whether the binary outcome described above of habit formation is associated with positive changes in behavioral automaticity using the SRBAI. Behavioral automaticity is the hypothesized key MoBC for this trial. A Fisher exact test (5%, 2-sided) will be used to examine whether an hourly walking habit formation will be associated with positive changes in behavioral automaticity. Specifically, for each participant, the difference between average behavioral automaticity during the last 2 weeks of intervention and average baseline behavioral automaticity will be calculated; a positive change is defined as a difference of greater than zero. Additionally, we will model the association between habit and continuous changes in automaticity over time using autoregressive models of order 1 to account for serial autocorrelation between repeated measures of the SRBAI. Finally, a mixed-effects logistic regression will be used to assess the effects of behavioral automaticity on the development of a daily walking habit, with adjustment for other factors, such as caregiver demographic characteristics and factors related to caregiving. For this model, we will examine the fixed effects of automaticity, time, and the time-by-automaticity interaction effect to examine whether changes in automaticity over time are associated with habit formation.

Additionally, another secondary hypothesis of this trial examines the association between longitudinal behavioral automaticity and habit formation over time. These analyses will provide preliminary estimates of how behavioral automaticity changes at a given week impact the attainment of the first operationalization of habit formation, thus informing how the timing of behavioral automaticity change will impact habit. Briefly, the association between habit formation and behavioral automaticity (measured by the SRBAI) at a given week will be assessed first using univariate logistic regression. Then, weekly behavioral automaticity changes will be jointly explored in a multivariable isotonic regression model, which will be analyzed using the R package *McMiso* [85,86]. Briefly, each participant’s achievement of habit formation (binary yes or no) at a given week will be regressed on SRBAI in that week and the week number using multivariable isotonic regression. This analysis will include data up to the first week where a habit is formed; if a habit is not formed, we will use SRBAI data in all 10 weeks. To further assess lagged effects, we will next regress achievement of habit formation on SRBAI in the same week and in the previous week, as well as the week number. A few methodological notes are in order. First, these analyses will include data up to the first week where a habit is formed. For example, if a habit is not formed in a participant, we will use SRBAI data for all 10 weeks. Once a habit is formed, we will not use SRBAI data after the event, so that temporally the dependent outcome is preceded by the independent variables. Second, isotonic regression assumes a monotonic association between habit (dependent variable) and the independent variables (SRBAI and time). This assumption is reasonable because automaticity (as measured by the SRBAI) should increase proportionally as habit formation becomes more likely. Also, since time as a covariate indicates the *cumulative* risk of forming a habit, we also expect the association between habit and week number to be positive. Third, isotonic regression does not impose additional assumptions other than monotonicity, such as linearity as in logistic regression and additivity between covariates, and hence represents a data-driven tool to explore the relationship between habit and SRBAI.

A final secondary outcome for the trial is examining HTEs for habit formation and on changes in behavioral automaticity by conducting analyses of HTEs across participants. This will involve examining the heterogeneity in time to attaining the first operationalization of habitual hourly walking due to the BCT intervention.

### Exploratory Outcomes

Exploratory analyses will be conducted to examine the important potential moderating variables that may influence the effect of the intervention on habitual hourly walking. These include caregiver sociodemographic characteristics from a survey required by the funder (eg, sex at birth, gender identity, sexual orientation, race, ethnicity, highest education level, household income level, occupation category, current marital status, living situation, insurance status, and zip code) and self-reported details of caregiving (eg, number of care recipients, relation to person with ADRD, primary caregiver status, length of time providing care, hour providing care per week, living situation, time since diagnosis, and demographic characteristics of person with ADRD). Participants will complete pre- and postintervention measures to assess various components of caregiver well-being, including (1) burden using the Zarit Burden Interview Short Form (12 items), which uses a rating scale of 0 (never) to 4 (nearly always) with a higher total score equating to higher burden [64], (2) stress using the PSS-10 validated in English and Spanish with a rating scale of 0 (Never) to 4 (Very Often) with a higher total score equating to higher stress [65,66]; (3) anxiety symptoms using the STAI-S with 2 scales, the State anxiety and Trait anxiety scale, both with a scale of 0 (not at all) to 3 (very much so) with a higher total score equating to higher anxiety symptoms [67]; (4) depressive symptoms using the adapted PHQ-9; excluding the final question assessing thoughts of suicide or harming oneself) with a 4-point Likert scale from 0 (not at all) to 3 (nearly every day) with a higher total score equating to higher depressive symptoms [68]; and (5) quality of life using the EuroQol-EQ-5D-5L with a level score of 1 (indicating no problem) to 5 (indicating unable to or extreme problems) for 5 health state items and a 0 (worst health you can imagine) to 100 (best health you can imagine) visual rating scale for overall current health converted into an index value [69]. The EuroQol-EQ-5D-5L official Spanish translation was obtained from the EuroQol Research Foundation, which follows a standardized translation protocol that involves a forward and backward translation process [70,71]. Additionally, participants will complete preintervention measures of the NPIQ, a brief informant-report of symptom severity and associated caregiver distress for 12 neuropsychiatric domains, and the Katz ADLs, a binary checklist assessing the functional independence or dependence of older adults in performing 6 basic ADLs [72-75].

### Sample Size Calculation

A sample of 100 participants is planned for enrollment into this remote trial. Up to 20% attrition is expected following the 2-week baseline period due to failure to meet wearable device and survey adherence requirements (n=20). An additional 25% attrition is expected after the 10-week intervention period due to participants becoming lost to follow-up, withdrawing, or other (n=20). The expected attrition throughout the intervention period is estimated as such because of the burdens caregivers experience due to the nature of their unique responsibilities, not limited to time constraints and motivation, which may produce barriers to longitudinal research participation [87]. We have attempted to design this trial to minimize barriers to participation for caregivers specifically, yet have estimated attrition similar to that in other longitudinal physical activity trials with caregivers to be conservative [88,89].

The trial still will have a sufficient sample (n=60) to provide adequate power. Assuming a null habit formation rate of 30% with a sample size of n=60, a 1-sided, one-sample binomial test at 5% significance will have about 92% power to declare the BCT intervention effective if the true habit formation rate is at 50% or above. We believe the null hypothesis for habit formation is conservative, as caregivers spend 80% of their time being sedentary.

To anticipate the variability due to the proposed total sample size of 60, we calculated the power to detect a difference between 20% habit formation (in the group of participants without increased behavioral automaticity) versus 60% habit formation (in the group of participants with increased behavioral automaticity) under different scenarios. Specifically, when 30% of the participants experience an increase in behavioral automaticity, there is 86% power to detect the difference. When 50% of the participants experience increases in behavioral automaticity, the power increases to about 89%.

## Results

### Recruitment Status

This National Institute on Aging–funded trial was awarded in June 2024. The trial is designed to enroll participants on a continuous, rolling basis. Recruitment and data collection began in March 2025. Forty ADRD caregiver participants (40% of the planned sample) have been enrolled as of August 2025. No major deviations from the original protocol have occurred. We expect to complete data collection by June 2026. Data analysis of primary, secondary, and exploratory outcomes is expected to be complete by Fall 2026. Deidentified raw datasets along with data dictionaries, and the trial protocol and statistical analysis plan will be posted on the trial’s Open Science Framework page at the time of publication of results. We expect to publish results by Winter 2026. Results will be posted with the trial registration on ClinicalTrials.gov in accordance with policy.

## Discussion

### Principal Results

This NIH Stage II decentralized behavioral trial has the potential to advance science on forming a habit to disrupt sedentary behavior among caregivers for persons with ADRD and will help determine whether behavioral automaticity acts as the primary MoBC for the effect on this BCT intervention to form an hourly walking habit. If successful, this trial will inform further NIH Stage III effectiveness trials, can be applied to other ADRD populations, and can be applied to other physical activity behaviors, such as moderate activity interventions. Although out of scope for this trial, additional areas of exploration should also include objective confirmation of sedentariness and maintenance of habit formation to understand whether hourly walking habits persist long-term and beyond delivery of the intervention.

### Potential Limitations

A limitation of this trial includes possible issues with the generalizability of the study findings. Based on the demographic makeup of caregivers of people with ADRD across the United States, a majority female sample is expected [7]. A second limitation is that this trial was not designed to capture nonwalking sedentary interruptions, such as periods of standing, by nature of the research objective, and the use of Fitbit wearable devices to collect continuous data. While there is some evidence that disrupting prolonged sitting behavior with standing has positive health associations, the positive health outcomes associated with disrupting sedentary behavior with walking are much more demonstrated and understood in the literature, hence the focus of this efficacy trial [90-94]. A third limitation is that we identified behavioral consistency for hourly walking (eg, walking 250 steps per h for 4 consecutive h and for 7 days in a row) as a definition for our primary outcome of habit formation. This metric was used to allow us to use a dichotomous measure of habit formation while also capturing the time duration required to achieve formation of habit. However, multiple additional criteria could easily be used to determine the presence or absence of habitual behavior. We will also be measuring daily steps longitudinally over the full duration of the trial to address this. Additional limitations may be with SMS text message fatigue, intervention fatigue, and/or low participant engagement, all of which could subsequently lead to missing data, as caregivers are a population who already experience burden and so may have barriers to completing the trial successfully. Our trial is intentionally designed with caregivers’ unique needs in mind (eg, no in-person study visits, use of electronic survey data capture, and personalized intervention times). We also intentionally designed the trial to deliver the daily components of the BCT intervention using concise language and as behavioral “nudges” that do not require engagement (eg, a response) from the participant to reduce perceived messaging or intervention fatigue. Yet, it may still be that participation is not feasible for all caregivers. Strategies will be implemented to alleviate perceived challenges of participating in the research throughout the trial, including weeknight and weekend clinical research staff support for caregivers who may only be available outside of standard business hours, providing caregivers the option to pause participation due to unanticipated circumstances and resume at a better time, and, if the caregiver wishes to withdraw from the research, offering caregivers the option to partially withdraw by passively providing only Fitbit data (rather than fully withdraw). Additionally, we have statistical methods and sensitivity analyses to manage missing data as described in the Methods section.

### Conclusions

This trial represents an opportunity for advancement in knowledge about the effectiveness of forming a habit using BCTs to disrupt sedentary behavior in caregivers of persons living with ADRD, and “how” they work via the hypothesized MoBC behavioral automaticity. Based on the literature, remotely delivered, easily administered behavior interventions focusing on habit formation have the potential to create behavior change, and trial designs intended to minimize perceived challenges may provide opportunities for future effectiveness trials to create public health impact to promote physical activity.

## Abbreviations

ADL: activity of daily living
ADRD: Alzheimer disease and related dementias
BCT: behavior change technique
HIPAA: Health Insurance Portability and Accountability Act
HTE: heterogeneity of treatment effect
IRB: Institutional Review Board
ISO: International Organization for Standardization
MoBC: mechanism of behavior change
NIH: National Institutes of Health
NPIQ: Neuropsychiatric Inventory-Questionnaire
PHQ-9: Patient Health Questionnaire-9
PSS-10: Perceived Stress Scale
REDCap: Research Electronic Data Capture
SRBAI: Self-Report Behavioral Automaticity Index
STAI-S: State-Trait Anxiety Inventory – Short Form
